# Gravi-D peptide disrupts HDAC11 association with an AKAP to stimulate adipocyte thermogenic signaling

**DOI:** 10.1172/JCI177726

**Published:** 2024-05-01

**Authors:** Emma L. Robinson, Charles A. Tharp, Rushita A. Bagchi, Timothy A. McKinsey

**Affiliations:** 1 Department of Medicine, Division of Cardiology and; 2 Consortium for Fibrosis Research & Translation, University of Colorado Anschutz Medical Campus, Aurora, Colorado, USA.

**Keywords:** Cell biology, Therapeutics, Adipose tissue, Signal transduction

**To the editor:** Inhibition of the lysine demyristoylase activity of histone deacetylase 11 (HDAC11) induces adipocyte protein kinase A (PKA) signaling and expression of uncoupling protein 1 (UCP1), which promotes nonshivering thermogenesis and energy expenditure (1). PKA/UCP1 induction upon HDAC11 inhibition requires enhanced lysine myristoylation and lipid raft (LR) targeting of the HDAC11 substrate, A-kinase anchoring protein 12 (AKAP12, also known as gravin-α). Gravin-α is 1 of only 2 known myristoyl substrates of HDAC11; studies in nonadipocytes showed that HDAC11 is also capable of demyristoylating serine hydroxymethyltransferase 2 (SHMT2) to regulate interferon signaling (2). The consequences of augmenting myristoylation of other, as-yet-unidentified adipocyte targets of HDAC11 upon general catalytic inhibition of the enzyme remain undetermined.

To circumvent this potential issue, we sought to develop an approach to selectively inhibit the pool of HDAC11 that associates with gravin-α. Our prior work defined a 50–amino acid HDAC11 binding domain adjacent to the PKA binding domain on gravin-α (3). Further mapping and coimmunoprecipitation studies delineated a conserved 10–amino acid HDAC11 binding domain on gravin-α (Figure 1, A and B). The conversion of 2 glutamines in this region to alanine (QQ/AA) dramatically reduced the association of HDAC11 with full-length gravin-α (Supplemental Figure 1A; supplemental material available online with this article; https://doi.org/10.1172/JCI177726DS1).

A peptide representing the 10–amino acid HDAC11 binding domain of gravin-α was fused to a glycine linker and an 11-arginine tail to yield a cell-permeable peptide referred to as gravin disruptor (Gravi-D; Figure 1C). To assess the potential of Gravi-D to disrupt HDAC11–gravin-α complexes and promote myristoylation of the anchoring protein, HEK293 cells were transfected with expression constructs encoding epitope-tagged versions of gravin-α and HDAC11 and treated with Gravi-D as well as a scrambled peptide or Gravi-D (QQ/AA), which served as negative controls (Supplemental Figure 1B). Gravi-D, but not the negative controls, reduced the amount of HDAC11 that coimmunoprecipitated with gravin-α, which correlated with enhanced myristoylation of the anchoring protein (Supplemental Figure 1, C–E). Gravi-D also stimulated gravin-α myristoylation in 3T3-L1 adipocytes, but failed to increase SHMT2 myristoylation in these cells, illustrating the specificity of the peptide for disruption of gravin-α–HDAC11 complexes (Supplemental Figure 2, A–C).

To address whether Gravi-D stimulates association of gravin-α with LRs, 3T3-L1 adipocytes were infected with lentiviruses encoding FLAG-tagged wild-type (WT) gravin-α or a myristoylation-deficient version of gravin-α (K1502/1505R), and treated with scrambled peptide, Gravi-D, or the pharmacological HDAC11 inhibitor, FT895, which served as a positive control (Figure 1D). Gravi-D was as effective as FT895 at promoting association of gravin-α with the canonical LR proteins caveolin-1 (Cav-1) and flotillin-2 (Flot-2), and these inducible interactions required gravin-α lysine myristoylation, since Gravi-D failed to trigger the association of gravin-α (K1502/1505R) with the LR proteins (Figure 1E).

To determine whether Gravi-D is capable of stimulating thermogenic signaling, 3T3-L1 adipocytes were treated with Gravi-D in the absence or presence of the β_3_-adrenergic receptor (β_3_-AR) agonist, CL-316,243 (CL), which served as a positive control. Remarkably, Gravi-D was a similarly effective inducer of PKA activation and UCP1 protein induction as CL (Figure 1F). Gravi-D also stimulated UCP1 expression in primary mouse adipocytes (Supplemental Figure 2D). We failed to observe an additive effect upon combined treatment with Gravi-D and the β_3_-AR agonist (Supplemental Figure 2E). Profound Gravi-D–mediated UCP1 induction was also revealed by immunostaining of adipocytes (Figure 1G).

In addition to stimulating UCP1 expression, PKA promotes adipocyte lipolysis via phosphorylation of hormone-sensitive lipase (HSL) (4). Gravi-D treatment of 3T3-L1 cells stimulated HSL phosphorylation at the activating PKA target sites and promoted adipocyte lipolysis, as determined by neutral lipid release into the cell culture medium (Supplemental Figure 3, A and B).

The inability of β_3_-AR agonists to consistently increase energy expenditure in humans has been partially attributed to catecholamine resistance, wherein chronic exposure to ligand and/or inflammatory cues results in a reduction in β_3_-AR expression and signaling (5). HDAC11 inhibition with FT895 circumvents catecholamine resistance in cell-based and in vivo models (1). To address the capacity of Gravi-D to also bypass catecholamine resistance, 3T3-L1 adipocyte were exposed to vehicle or CL for 20 hours prior to 1 hour of reexposure to these agents, the positive control, FT895, scrambled peptide, or Gravi-D (Supplemental Figure 4A). Chronically treating adipocytes with CL led to reduced β_3_-AR protein expression and blocked subsequent PKA and UCP1 induction in response to acute reexposure to this agonist, which is indicative of catecholamine resistance (Supplemental Figure 4B). Strikingly, Gravi-D was as effective as FT895 at bypassing β_3_-AR downregulation to induce PKA and UCP1 (Supplemental Figure 4B).

In summary, Gravi-D functions downstream of β-ARs to promote PKA-mediated thermogenic and lipolytic signaling independently of cell-wide inhibition of HDAC11 (Figure 1H). Our findings underscore the potential for using peptides or small molecules to disrupt HDAC11 binding to gravin-α as a means of increasing energy expenditure to treat obesity and metabolic disease.

## Supplementary Material

Supplemental data

Unedited blot and gel images

Supporting data values

## Figures and Tables

**Figure 1:**
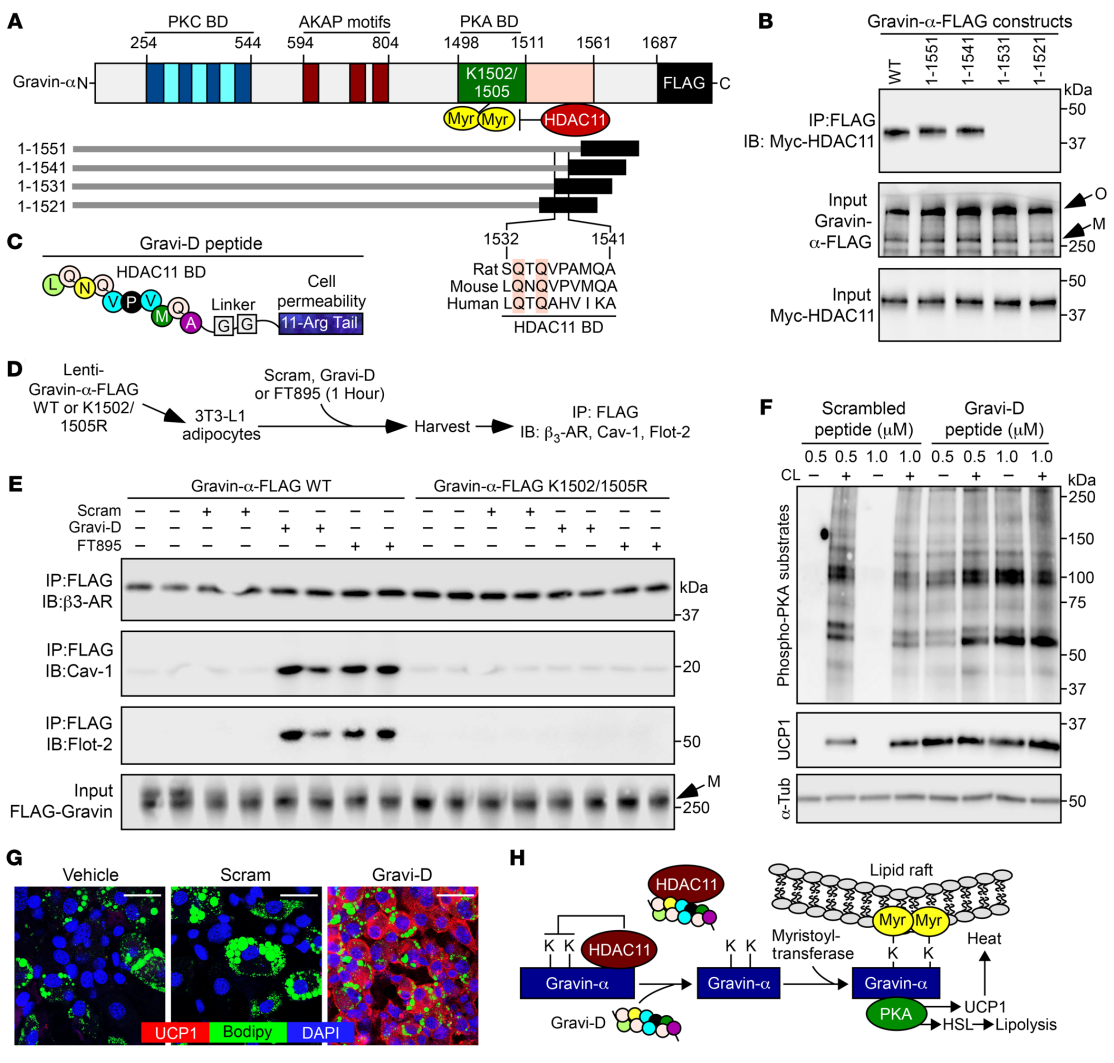
Gravi-D peptide stimulates adipocyte PKA signaling and UCP1 induction. (**A**) Schematic representation of full-length and truncated forms of rat gravin-α. The 10–amino acid HDAC11 binding domain is indicated. (**B**) Coimmunoprecipitation of Myc-tagged HDAC11 with full-length (WT) and truncated forms of gravin-α in transfected HEK293 cells; gravin-α migrates as a monomer (M) and oligomer (O) in SDS-PAGE. (**C**) Schematic depiction of Gravi-D. (**D**) Schematic depiction of the lipid raft protein coimmunoprecipitation study. (**E**) Coimmunoprecipitation analysis of the indicated proteins. (**F**) 3T3-L1 adipocytes were treated with vehicle control or CL-316,243 (1 μM) for 30 minutes followed by scrambled peptide or Gravi-D for 1 additional hour, and harvested for immunoblotting. (**G**) Immunofluorescence images of 3T3-L1 adipocytes treated with vehicle (water), scrambled peptide (1 μM), or Gravi-D (1 μM) for 1 hour. Scale bars: 50 μm. (**H**) A model for Gravi-D–mediated induction of thermogenesis and lipolysis. For all blots, each lane represents protein from an independent plate of cells.

## Version 1 05/01/2024

Electronic publication
