# Supplementary material for: Gravi-D peptide disrupts HDAC11 association with an AKAP to stimulate adipocyte thermogenic signaling
Source: J Clin Invest. 2024 May 1;134(9):e177726. doi: 10.1172/JCI177726 (PMC11060728; doi:10.1172/JCI177726)
Supplement: Supplemental data [file jci-134-177726-s135.pdf]

Supplemental Material

Methods

Plasmids and lentivirus generation

p3XFLAG-CMV-14 expression vector (Sigma-Aldrich) encoding rat gravin-α was a gift from Dr. Joseph Miano, Augusta University, U.S.A. This base vector was used to generate truncated constructs of gravin-α via PCR using specific primers. Site-directed mutagenesis reactions were performed to create p3XFLAG-CMV-14 encoding rat gravin-α (QQ/AA) using the QuickChange method (Agilent Technologies). Lentiviral constructs were generated by cloning complementary DNAs encoding rat Gravin-α WT or KK/RR into pLenti CMV Hygro DEST. Lentiviruses were generated by co-transfection of pLenti plasmids, psPAX2 and pMD2.G into L293 cells. The viral supernatant was collected 60 hours after transfection, filtered through a 0.45 μm syringe filter. Viral supernatant was supplemented with polybrene at 1 μl/mL (stock concentration 10mg/mL) and used to directly infect adipocytes. After 24 or 48 hours of infection, cells were used for experiments as indicated. psPAX2 and pMD2.G were gifts from Didier Trono (Addgene plasmids # 12260, #12259).

Peptides

Gravi-D, scrambled and QQ/AA peptides were synthesized by Synpeptide and resuspended in sterile water. Cells were treated at a final peptide concentration as indicated.

Cell culture

The 3T3-L1 CL-173™ preadipocyte cell line was purchased from ATCC. Cells were maintained and differentiated into mature adipocytes *in vitro* as previously described (1). Undifferentiated cells were seeded on culture dishes and grown to 80-90% confluency in DMEM supplemented with 10% newborn calf serum and 1X Penicillin-Streptomycin-L-Glutamine. Differentiation of 3T3-L1 cells was induced using DMEM supplemented with 10% fetal bovine serum, 1 μM dexamethasone, 0.5 mM 3-isobutyl-1-methylxanthine, 1 μg/mL insulin for 72 hr. Following this incubation, media was changed to adipocyte maintenance media (DMEM with 1μg/mL insulin, 10% FBS) and replenished every 48h for 8 days or until lipid droplet coverage was 80 – 90%, as assessed by brightfield microscopy. At this time, 3T3-L1 adipocytes were treated as per the experiments described.

Primary preadipocytes were obtained from the stromal vascular fraction (SVF) of inguinal white adipose tissue of WT male C57BL/6J mice, as previously described (1). IngWAT was dissected and washed in pre-warmed DMEM before being transferred to a petri dish containing 10 mL of 0.2% (w/v) collagenase II digestion buffer and subsequently cut into 1 mm^3 chunks and minced using spring scissors (Fine Science Tools). Minced ingWAT tissue buffer was transferred to a 15 mL Falcon tube in digestion buffer with the lid loosely fastened and incubated in a shaker at 37 ºC at 400 rpm for 20 minutes, with vortexing every 5 minutes. At the end of the digestion, the tissue slurry was passed through a 70 μm cell strainer into a 50 mL Falcon tube, followed by 30 mL preadipocyte media (5% NBCS, 5% FBS,1% PSG) to quench enzymatic activity. After a 2-minute) centrifugation at 200g at room temperature, the upper floating mature adipocyte-

containing layer was discarded. The cell suspension was then centrifuged at 2000 x g for 5 minutes at room temperature to pellet SVF-derived preadipocytes. The cell pellet resuspended in pre-warmed preadipocyte media. Cells were plated directly onto 6- well cell culture plates (VWR). Once 90 - 100% preadipocyte confluency was reached, differentiation was induced by treatment with differentiation induction medium (DMEM, 10% FBS, 0.5% PSG, 1 μg/mL insulin, 0.5 μM dexamethasone, 0.25 mM 3-isobutyl-1-IBMX and 1 um rosiglitazone) for 72 hours. On day 3, induction medium was replaced with maintenance media (DMEM, 10% FBS, 0.5% PSG, 1 μg/mL insulin). Maintenance media was replenished every 48 hours and maturation followed by observing lipid droplet coverage by brightfield microscopy, with 80-90% lipid droplet coverage considered fully differentiated. Animal work for isolation of adipocytes was conducted using a protocol approved by the Institutional Animal Care and Use Committee of the University of Colorado Anschutz Medical Campus, following appropriate guidelines and in accordance with the United States Public Health Service Policy on Humane Care and Use of Laboratory Animals.

Coimmunoprecipitation analysis

For studies with HEK293 cells, gravin-α and HDAC11 expression constructs were transfected using polyethylenimine (PEI), or cells were mock transfected with PEI only. Twenty-four hours after transfection, total protein homogenates were prepared in PBS lysis buffer (100mM NaCl, 0.5% Triton X-100, PBS pH 7.4 with protease and phosphatase inhibitors; HALT, Thermo Scientific, PI-78443) and protein concentrations were determined using a BCA Protein assay. 500 μg of protein was subjected to

immunoprecipitation with 25 μl of anti-FLAG magnetic beads (Thermo Fisher Scientific, A36797) overnight at 4°C with end-over-end rotation. Following incubation, beads were collected and washed three times, denatured in sample loading buffer, and resolved through SDS polyacrylamide gels. Whole-cell lysates were used as input controls. Proteins were transferred to nitrocellulose membranes (0.45 μm; Life Science Products) and immunoblotted. The membranes were blocked with 50% LICOR blocking buffer in TBS-Tween (TBS-T; 20 mM Tris, 150 mM NaCl, 0.1% Tween 20) and incubated with primary antibodies (unconjugated or HRP conjugated) in 25% LICOR blocking buffer TBS-T for 1 - 2 hours at room temperature or overnight at 4°C. Membranes were incubated with secondary antibodies (where required); Alexa Anti-Rabbit 594 secondary antibody (Thermo Scientific, A-11012), anti-Mouse HRP secondary antibody (Southern Biotech, OB1031-05), anti-Rabbit HRP secondary antibody (Southern Biotech, OB4050-05). Protein bands were visualized using enhanced chemiluminescence reagents on an Odyssey® XF digital imaging system (LI-COR) using the chemiluminescence program and LI-COR Acquisition software.

For studies with 3T3-L1 adipocytes, cells were infected with 1 mL crude supernatant containing lentiviruses encoding FLAG-tagged WT rat gravin-α or lysine-myristoylation inactive mutant form (K1502/1505R) for 24h prior to preparing protein homogenates for coimmunoprecipitation studies.

Indirect immunofluorescence

3T3-L1 cells were grown and differentiated on glass coverslips (25 mm diameter) in 6-well tissue culture plates. Cells were fixed with 4% formaldehyde in PBS for 20 minutes

at room temperature followed by permeabilization in 0.25% Triton X-100 in PBS for 15 minutes. Non-specific antigen binding was blocked by incubating the cells with 5% bovine serum albumin in PBS for 1 hour at room temperature. Immunostaining was performed by incubating UCP1 primary antibody (Abcam, ab10983) at a concentration of 1:500 in 2.5% BSA in PBS overnight at 4 ºC on a rocker. Cells were washed thrice with PBS and incubated with fluorescent secondary antibody (Thermo Scientific, A-11012; 1:500) along with BODIPY (5 mM) and 4',6-diamidino-2-phenylindole (DAPI) at a final concentration of 300 nM in PBS for one hour at room temperature, rocking gently. Cells were then washed twice in PBS and mounted onto white frosted microscope slides. Imaging was performed on a Zeiss LSM780 confocal microscope (Advanced Light Microscopy Core, University of Colorado Anschutz Medical Campus) using a 40x objective lens, and images were processed through ZEN Black software (Zeiss).

Click chemistry

Detection of lysine myristoylation on gravin-α or SHMT2 was performed using a Click chemistry approach. Cells were supplemented with 50 μM alkynyl myristic acid (Click Chemistry Tools, 1164) for 4 hours prior to harvest, then lysed in 1% SDS lysis buffer (50 mM Tris-HCl pH 8.0, 1% (w/v) SDS) with protease and phosphatase inhibitors. Cell lysates were prepared as standard, and protein concentration was determined using a BCA Protein Assay Kit. Click chemistry reactions with Azide-PEG3-FLAG (Sapphire North America, CLK-032-S; 50 μM final concentration) were performed at room temperature for 30 minutes with end-over-end rotation with up to 200 μg of protein per sample. To precipitate and concentrate proteins, 600 μL of methanol, 150 μL of chloroform and 400

μL of 18 MΩ water were added to each sample. Samples were vortexed and centrifuged at 13,000 x g for 5 minutes at room temperature. The upper aqueous supernatant was gently removed and the interface layer containing the protein precipitate was left intact. 450 μL of methanol was added to the tubes, vortexed, and the samples spun at 13,000 x g for 5 minutes to pellet the protein. The methanol supernatant was removed, and the protein pellets were washed once again with 450 μL of methanol or until the protein pellets were white in color. The protein pellets were then air-dried for 15-30 minutes and resolubilized in 1% SDS buffer. Protein concentration was assessed by BCA assay, and 100 μg of FLAG-labeled protein was then incubated with 50 μL of anti-FLAG magnetic beads overnight at 4°C with end-over-end rotation. Following incubation, beads were collected with a magnetic rack and rinsed three times with TBS-T washing buffer (Tris-buffered saline containing 0.1% Tween™-20). After the final wash, the supernatant was discarded and 50 uL 2X sample buffer (100 mM Tris pH 6.8, 4% SDS w/v, 20% glycerol v/v, 10% β-mercaptoethanol and bromophenol blue) was added to the beads and boiled at 95 °C for 10 minutes. The samples were further analyzed via immunoblotting using antibody specific for gravin-α (Proteintech, 25199-1-AP; 1:1000) or SHMT2 (Santa Cruz Biotechnologies, sc-390641; 1:500).

Immunoblotting

Cultured cells were washed with PBS and lysed with RIPA buffer (50mM Tris.HCl pH 8.0, 150mM NaCl, 1% NP-40, 0.5% sodium deoxycholate and 0.1% SDS supplemented with protease and phosphatase inhibitors) for 30 minutes on ice followed by scraping and centrifugation at 13,000 x g for 5 minutes. Protein concentrations were measured using

a BCA assay, and 10 - 30 μg of protein was resolved through 4 - 15% pre-cast polyacrylamide gels and transferred onto 0.45 μm nitrocellulose membranes. Membranes were blocked in 50% LICOR blocking buffer in TBS-T and proteins were analysed by immunoblotting using antibodies against specific antigens. Membranes were incubated with primary antibodies at a final concentration of 1:1000 in 25% LICOR blocking buffer in TBS-T for 1-2 hours at room temperature or overnight at 4ºC on a rocker. Washes were performed thrice for 10 minutes in TBS-T followed by secondary antibody incubation with horse radish peroxidase (HRP)-conjugated anti-rabbit or anti-mouse secondary antibodies at a final concentration of 1:2000 in 5% non-fat milk in TBS-T. Final washes were performed in 1 X TBS-T, thrice for 10 minutes, prior to imaging. Immunoblot images were acquired on an Odyssey® XF digital imaging system (LI-COR) using the chemiluminescence program and LI-COR Acquisition software. In addition to the gravin-α and SHMT2 antibodies listed above, immunoblotting was performed with anti-GAPDH (Proteintech, 60004-1: 1:2000), anti-α-tubulin (Santa Cruz Biotechnology, 5286; 1:1000), anti-β_3-AR (Abcam, ab94506; 1:1000), anti-phospho-PKA substrates (Cell Signaling Technology, 9624; 1:1000), anti-HSL (Cell Signaling Technology, 18381, 1:1000), anti-HSL phospho-Ser-563 (Cell Signaling Technology, 4139; 1:1000), anti-HSL phosphor-Ser-660 (Novus, NBP3-05459; 1:1000), and FLAG-HRP (Sigma, A8592; 1:1000), anti-Myc (Santa Cruz Biotechnology, 9E10; 1:1000), anti-caveolin-1 (Cell Signaling Technology, 3267; 1:1000) and anti-flotillin-2 (Cell Signaling Technology, 3436; 1:1000).

Lipolysis assay

Cell culture media from mature 3T3L1 adipocyte was harvested following treatment with vehicle (water), scrambled or Gravi-D peptide or CL-316,243 at 1 μM for 1 hr. Levels of neutral lipid (primarily, glycerol) released in the media were analyzed using a lipolysis assays, according to the manufacturer's instructions (Abcam, ab242307). Briefly, 40 μl of lipid standard (3.9 – 500 mg/dL) or media sample was added to each well of a 96-well plate. 100X Fluorometric Reagent was diluted 1:100 with water and 200 μl added to each well of lipid standards and samples, incubated for 15 minutes at room temperature, protected from light. The plate was read on a Biotek microplate reader at 490 nm excitation and 585 nm emission. Neutral lipid levels in the adipocyte media were calculated according to the standard curve. For graphs, data are presented as mean +SEM, with vehicle treatments set to 1 in Supplemental Figures 1D and 1E, and CL treatment set to 1 in Supplemental Figure 2E. Statistical significance ($P < 0.05$) was determined using a one-way ANOVA with correction for multiple comparisons via a Tukey post-hoc test (GraphPad Prism 9).

Author contributions

E.L.R., R.A.B. and T.A.M. conceived and designed the study. E.L.R., R.A.B. and C.A.T. acquired, analyzed and interpreted data. E.L.R. and T.A.M. wrote and reviewed the manuscript. T.A.M. supervised the study. The order of the first or last authors reflects the leadership exerted in the study.

Acknowledgments

We thank J. Miano for the rat gravin-α cDNA construct and T. Hu for assistance with construct preparation. E.L.R. was funded by the American Heart Association (Grant 829504) and acknowledges support from the Colorado Clinical and Translational Sciences Institute (CCTSI, Project #CO-J-22-9) and Colorado Nutrition and Obesity Research Center (Project # YR29 25N216). R.A.B was supported by the Canadian Institutes of Health Research (Grant FRN-216927). R.A.B is currently supported by a Career Development Award from American Heart Association (23CDA1048663), Sturgis Endowment Grant for Diabetes Research, Brunson Pilot Award for Cardiovascular Research (UAMS), and a seed grant from the Arkansas Biosciences Institute. C.A.T. was supported by the NIH by grant HL007822. T.A.M. received funding from National Institute of Health by grants HL116848, HL147558, DK119594, HL127240, HL150225, and a grant from the American Heart Association (16SFRN31400013). Contents are the authors' sole responsibility and do not necessarily represent official NIH views**.**

1. Bagchi RA, Robinson EL, Hu T, Cao J, Hong JY, Tharp CA, et al. Reversible lysine fatty acylation of an anchoring protein mediates adipocyte adrenergic signaling. *Proc Natl Acad Sci U S A.* 2022;119(7).

Supplemental Figure 1

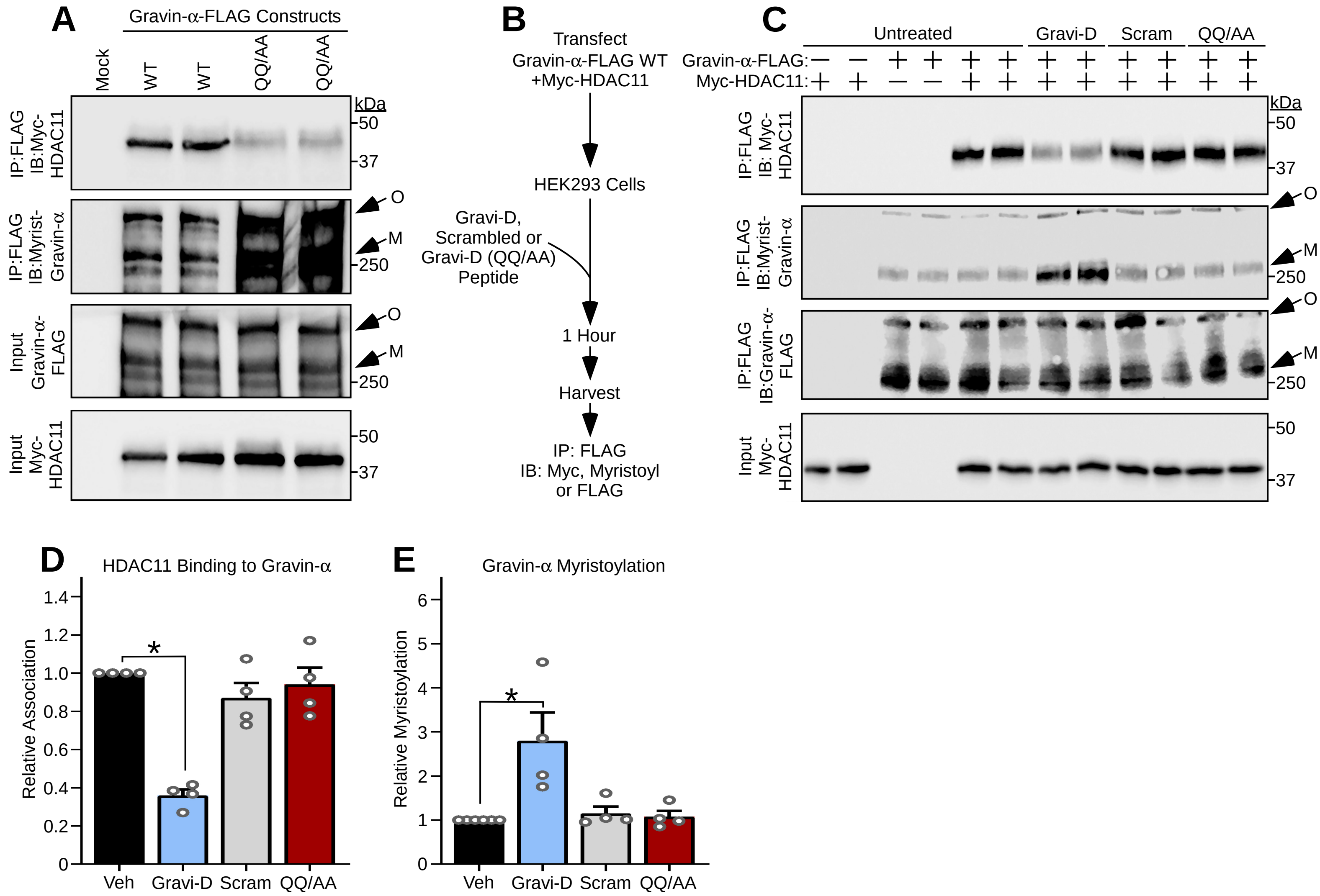

Supplemental Figure 1. Gravi-D peptide disrupts the association of HDAC11 with gravin-α. (A) HEK293 cells were transfected with expression constructs for FLAG-tagged wildtype (WT) rat gravin-α or Q1533/1535A (QQ/AA) and Myc-tagged HDAC11. Coimmunoprecipitation analysis revealed reduced binding of gravin-α QQ/AA to HDAC11. Gravin-α runs as a monomer (M) and an oligomer (O) in SDS-PAGE. (**B**) Schematic depiction of the experiment to test Gravi-D. (**C**) Coimmunoprecipitation analysis demonstrated that Gravi-D, but not scrambled peptide or QQ/AA peptide, reduced binding of HDAC11 to gravin-α, which correlated with enhanced myrisotylation of gravin-α. Total levels of gravin-α and HDAC11 were not changed by the treatments. Quantification of Gravi-D-mediated disruption of HDAC11 association with gravin-α (**D**) and gravin-α myristoylation (**E**) from the blots above, as well as an independent experiment. Each n represents an independent plate of cells. **P*<0.05 based on one-way ANOVA with Tukey's multiple comparisons test.

Supplemental Figure 2

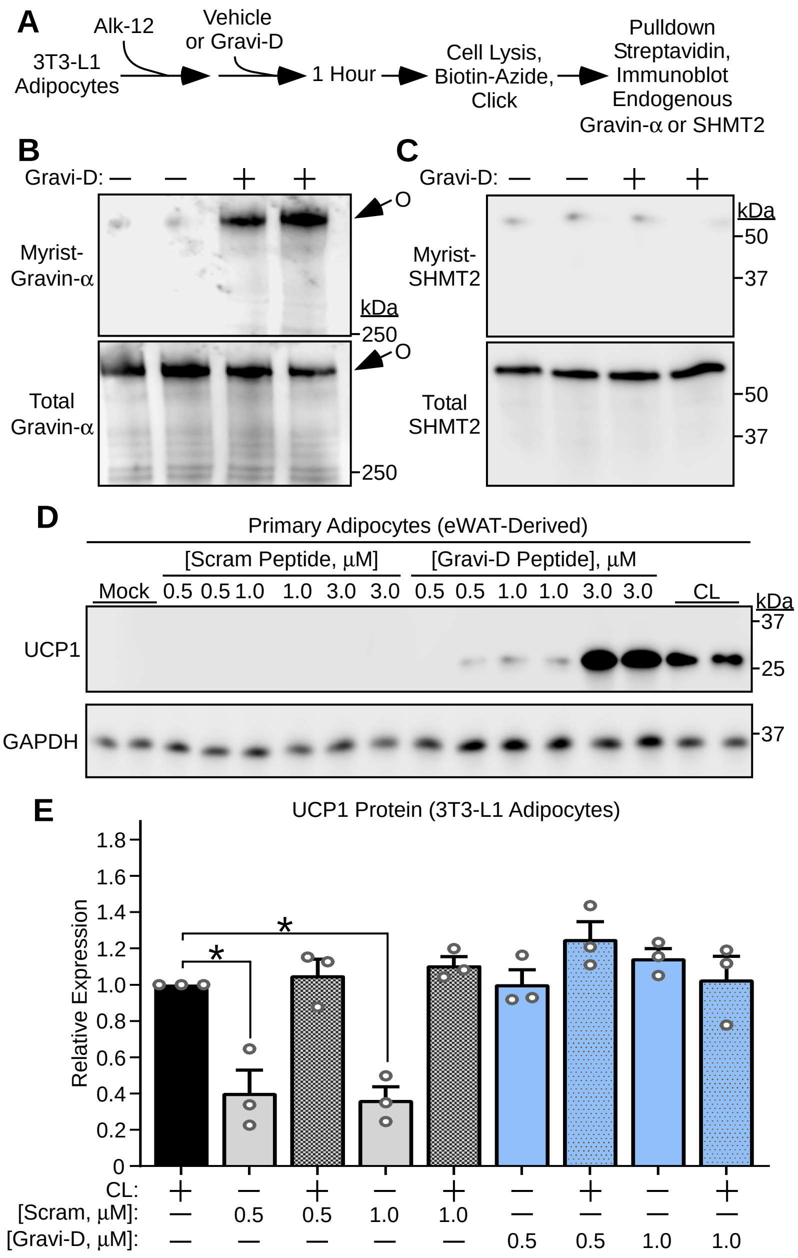

Supplemental Figure 2. Gravi-D stimulates gravin-α myristoylation and UCP1 expression in adipocytes. (**A**) Click chemistry approach to determine whether Gravi-D promotes myristoylation of gravin-α and/or SHMT2 in adipocytes. (**B**) Immunoblot analysis of myristoylated and total gravin-α; only the oligomeric form (O) of gravin-α was detected in this experiment. (**C**) Immunoblot analysis of myristoylated and total SHMT2. (**D**) Primary preadipocytes were purified from mouse epididymal white adipose tissue (eWAT) and differentiated into mature adipocytes in culture. Cells were treated with vehicle control (Mock), the indicated concentrations of scrambled or Gravi-D peptide, or CL-316,243 (1 μM) for 1 hour, and harvested for immunoblotting with the indicated antibodies. **(E)** Quantification of UCP1 expression in the blot shown in Figure 1F, as well as from two independent experiments. Each n represents a separate plate of cells; *$P<0.05$ based on one-way ANOVA with Tukey's multiple comparisons test.

Supplemental Figure 3

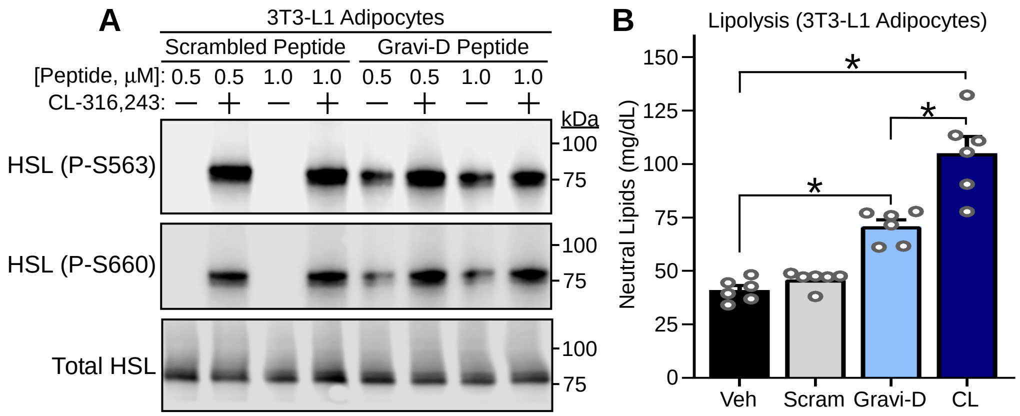

Supplemental Figure 3. Gravi-D stimulates adipocyte lipolysis. (A) 3T3-L1 adipocytes were pretreated with CL-316,243 (CL; 1 μM) or vehicle control (water) for 30 minutes and were subsequently exposed to scrambled or Gravi-D peptide for 1 hour. Immunoblot analysis was performed to assess phosphorylation of the PKA target sites on HSL. (**B**) 3T3-L1 adipocytes were treated for 1 hour with vehicle control (water), scrambled peptide (1 μM), Gravi-D (1 μM) or CL (1 μM) prior to quantifying lipolysis. Each n represents a different plate of adipocytes. *P<0.05 based on one-way ANOVA with Tukey's multiple comparisons test.

Supplemental Figure 4

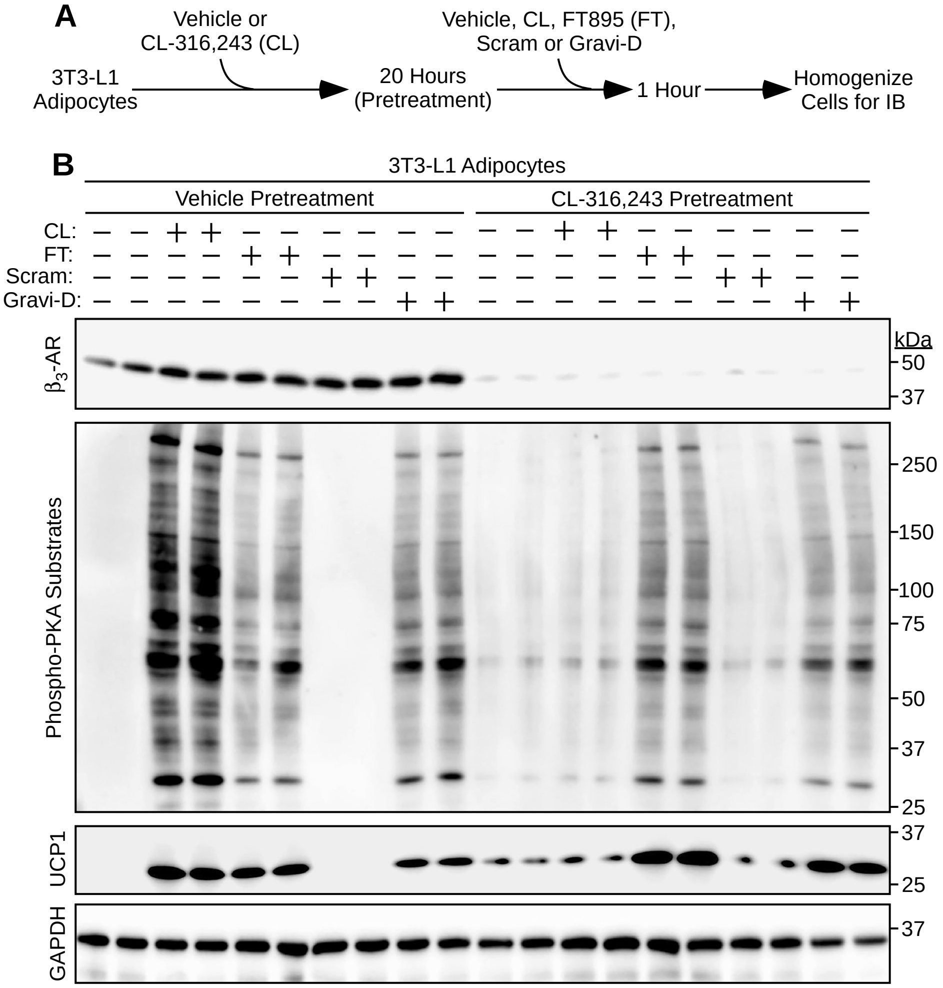

Supplemental Figure 4. Gravi-D promotes PKA activation and UCP1 induction in the context of catecholamine resistance. (A) Schematic representation of the cell culture experiment to determine whether Gravi-D promotes adipocyte PKA signaling and UCP1 expression in the context of downregulated β_3-adrenergic receptor (β_3-AR) expression due to chronic agonist exposure. (**B**) Immunoblot analysis of 3T3-L1 adipocytes pretreated with vehicle or the CL-316,243 (CL; 1 μM) for 20 hours followed by 1-hour treatment with DMSO vehicle (0.1% final concentration), CL (1 μM), FT895 (FT; 10 μM), scrambled peptide (1 μM) or Gravi-D (1 μM); n= 2 technical replicates/condition.
