# Supplementary material for: Gravi-D peptide disrupts HDAC11 association with an AKAP to stimulate adipocyte thermogenic signaling
Source: J Clin Invest. 2024 May 1;134(9):e177726. doi: 10.1172/JCI177726 (PMC11060728; doi:10.1172/JCI177726)

Fig. 1B

IP: FLAG, IB: Myc-HDAC11

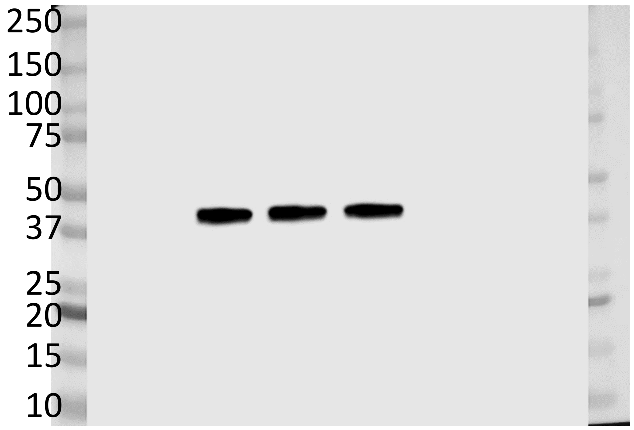

Input Gravin-α FLAG
and Input Myc-HDAC11

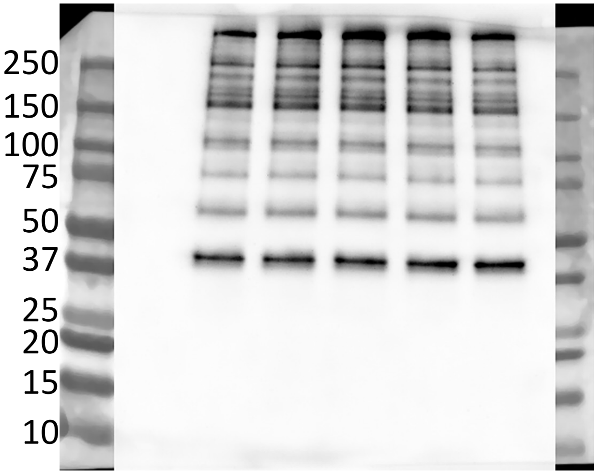

Fig. 1E

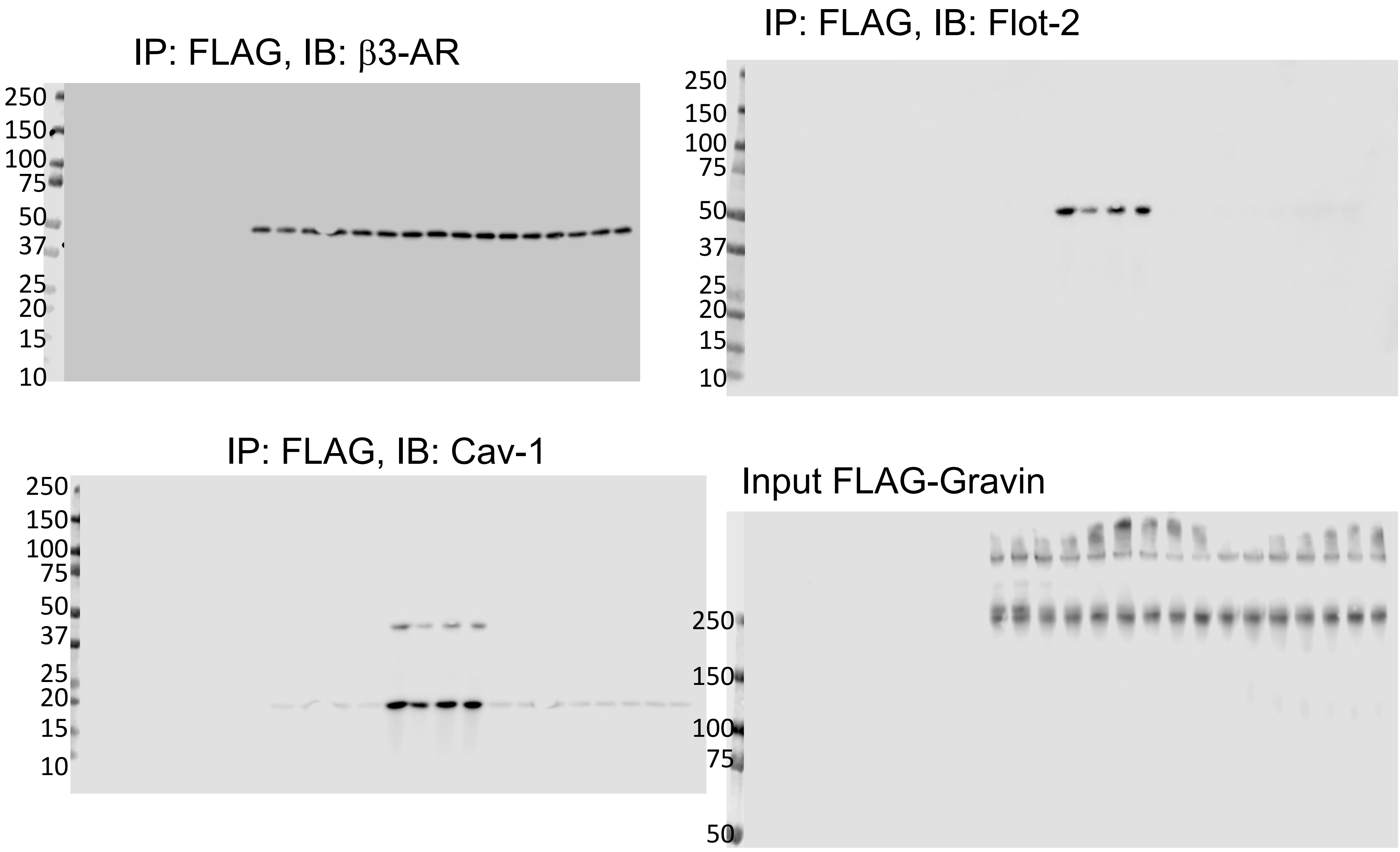
IP: FLAG, IB: β3-AR
250
150
100
75
50
37
25
20
15
10
IP: FLAG, IB: Flot-2
250
150
100
75
50
37
25
20
15
10
IP: FLAG, IB: Cav-1
250
150
100
75
50
37
25
20
15
10
Input FLAG-Gravin
250
150
100
75
50

Fig. 1F

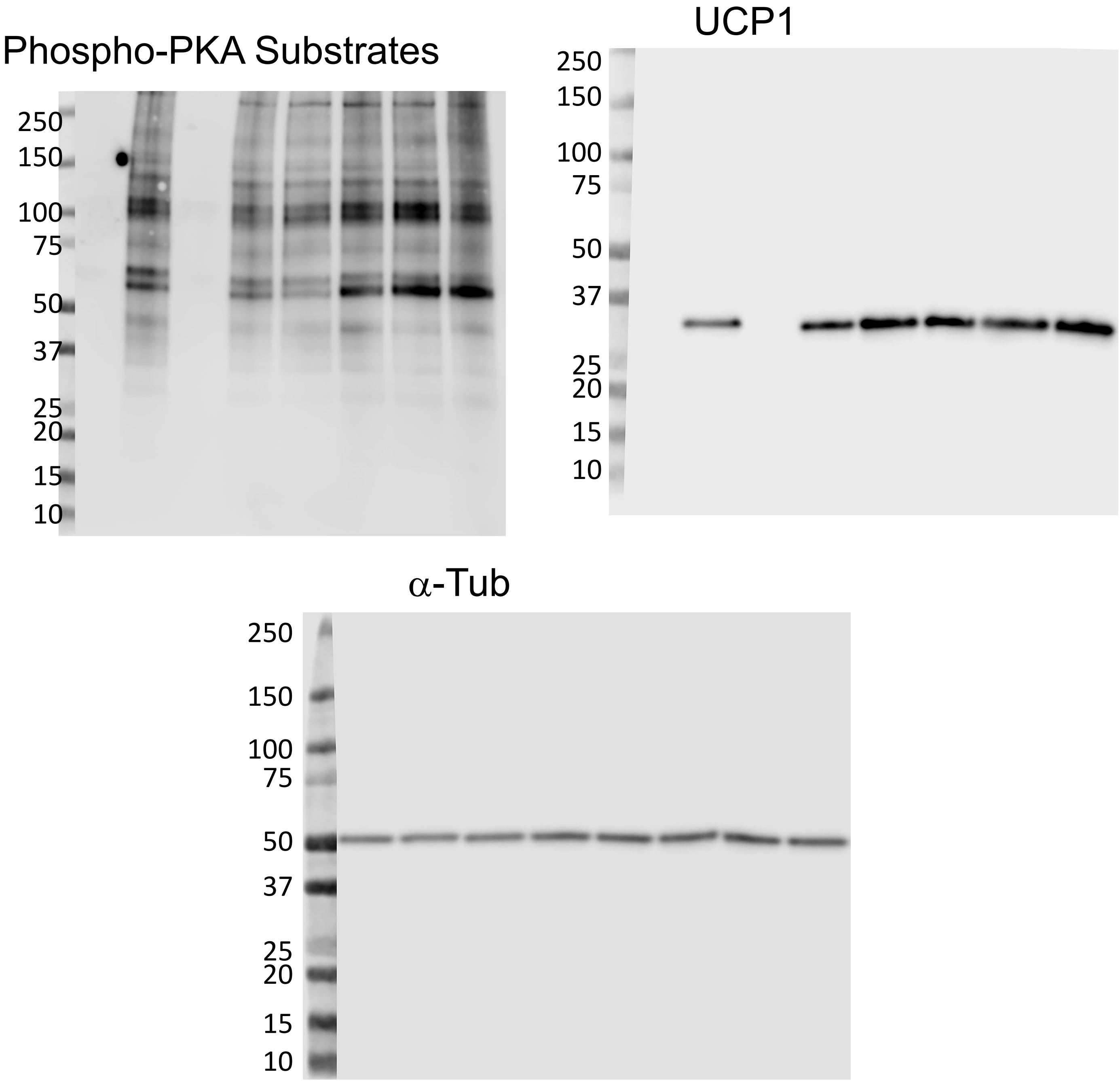
Phospho-PKA Substrates
250
150
100
75
50
37
25
20
15
10
UCP1
250
150
100
75
50
37
25
20
15
10
α-Tub
250
150
100
75
50
37
25
20
15
10

Supplemental Figure 1A

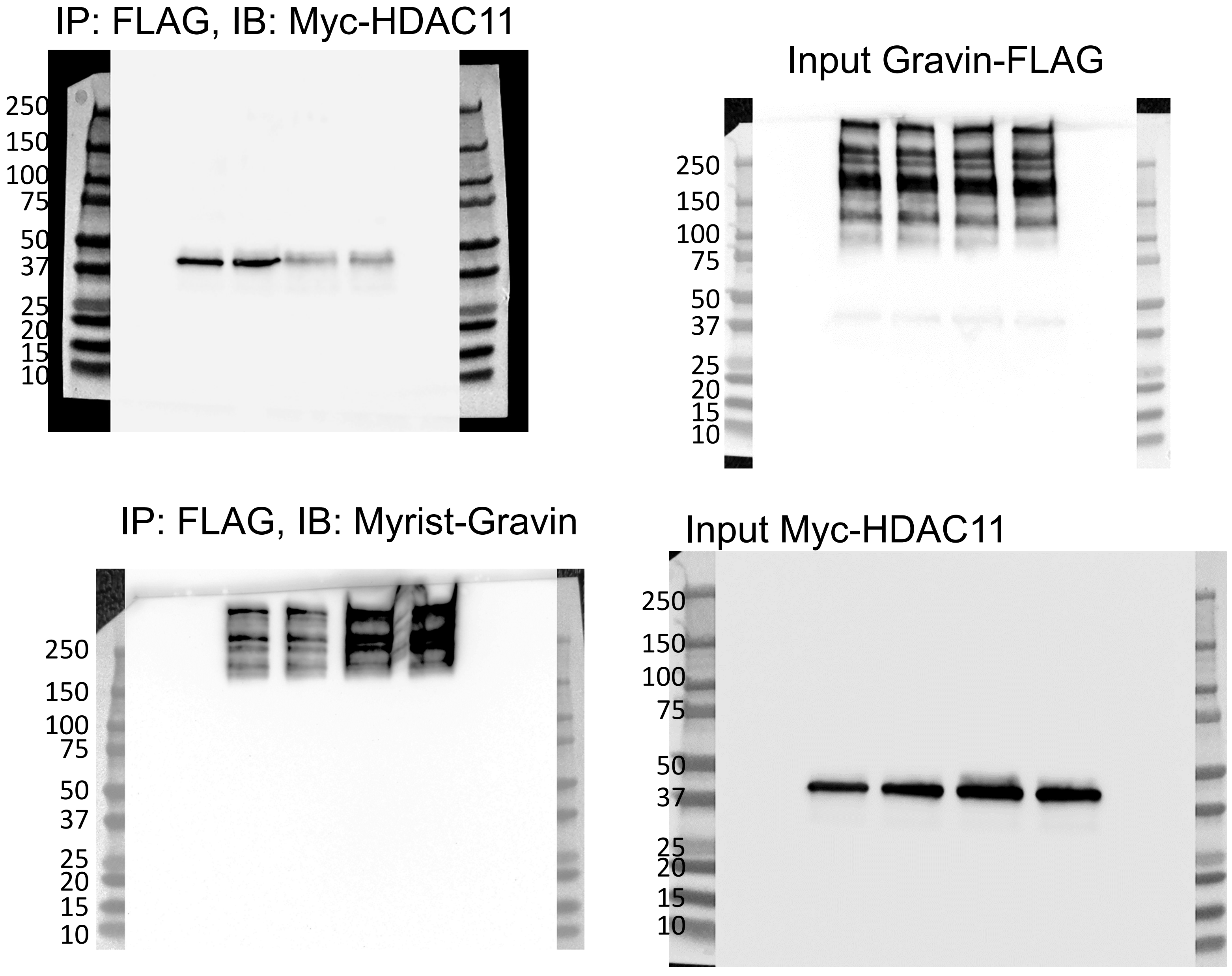

Supplemental Figure 1C

Membrane cut after Gravin-FLAG blot and reprobed for Myc

IP: FLAG, IB: Gravin-FLAG

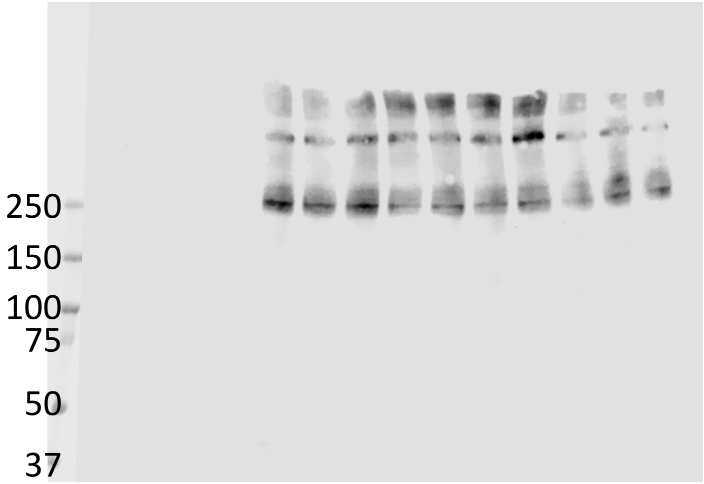

IP: FLAG, IB: Myc-HDAC11

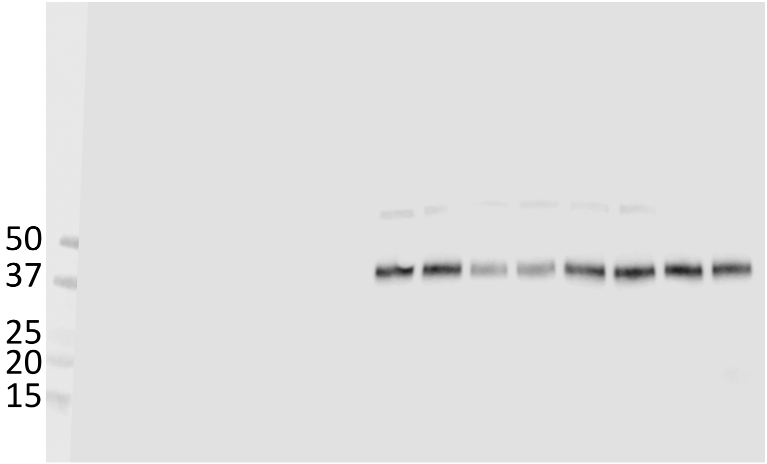

Lower half of membrane cut to avoid IgG signal

IP: FLAG, IB: Myrist-Gravin

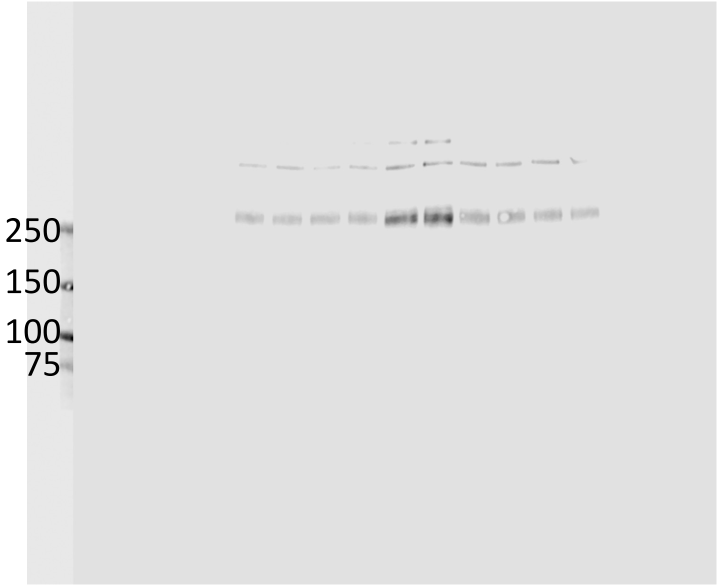

Input Myc-HDAC11

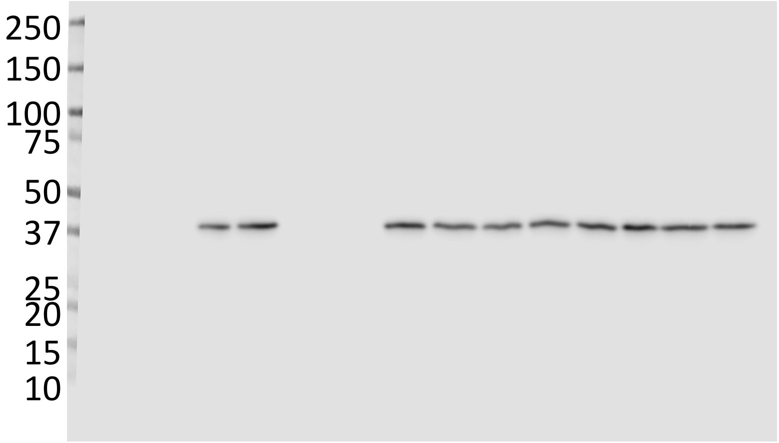

Supplemental Figure 2B and 2C

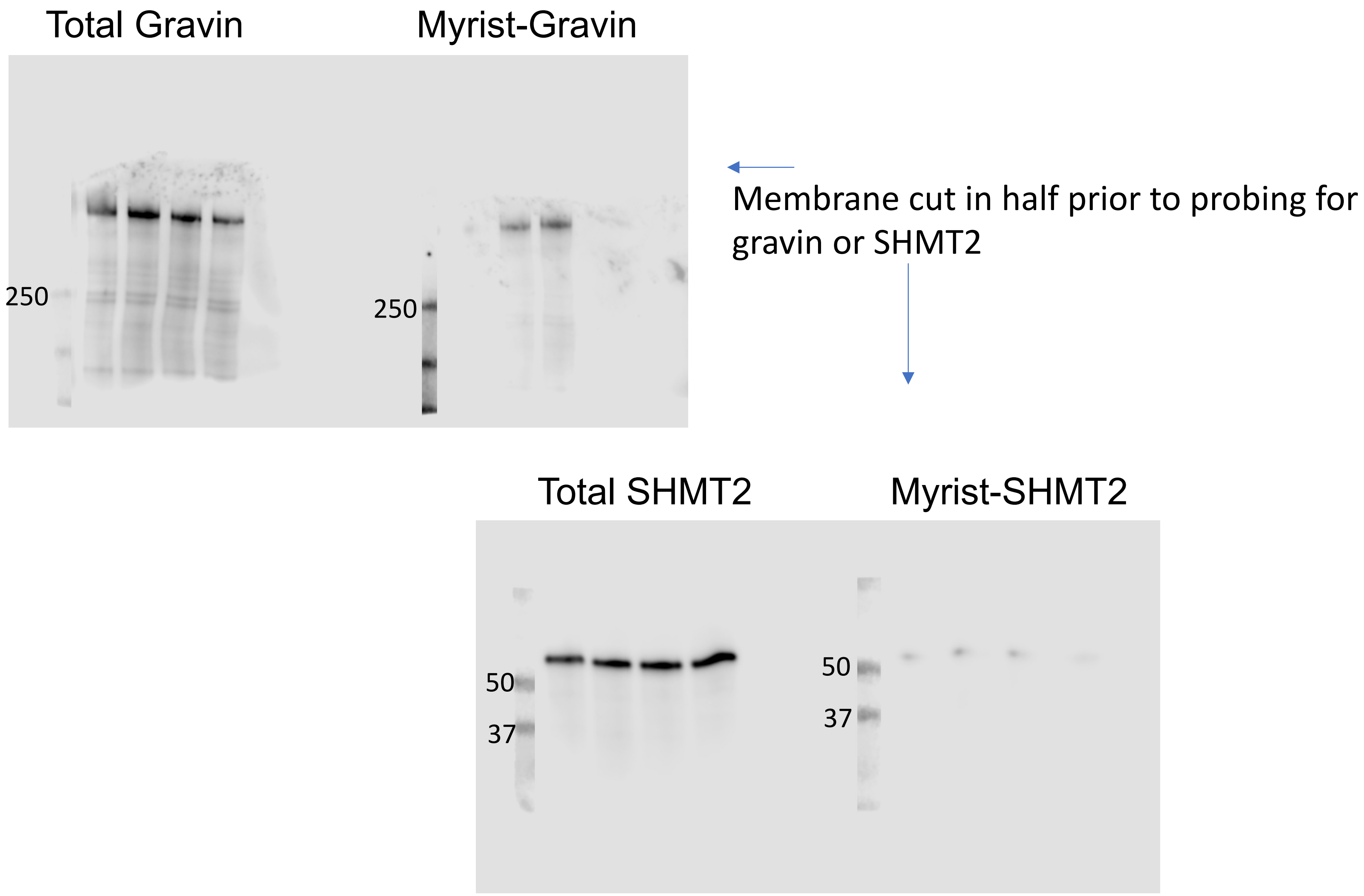

Supplemental Figure 3A

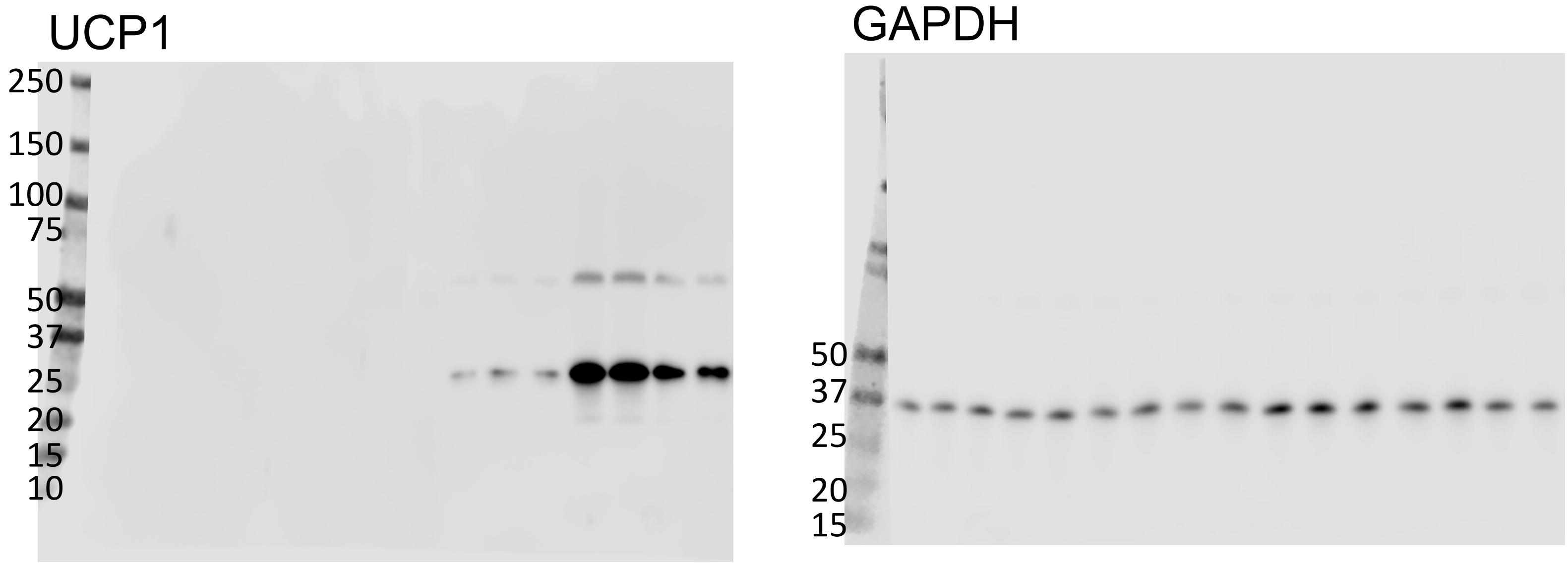

Supplemental Figure 4A

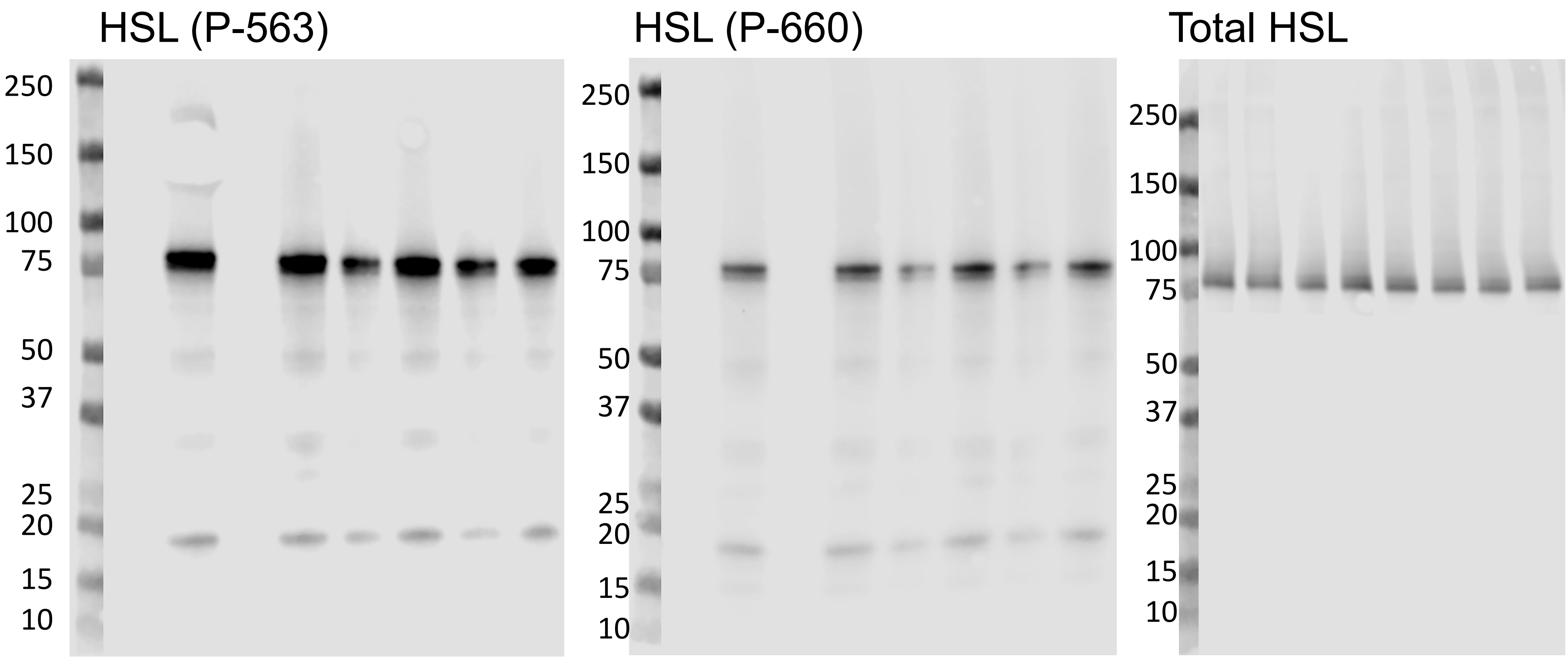

Supplemental Figure 5B

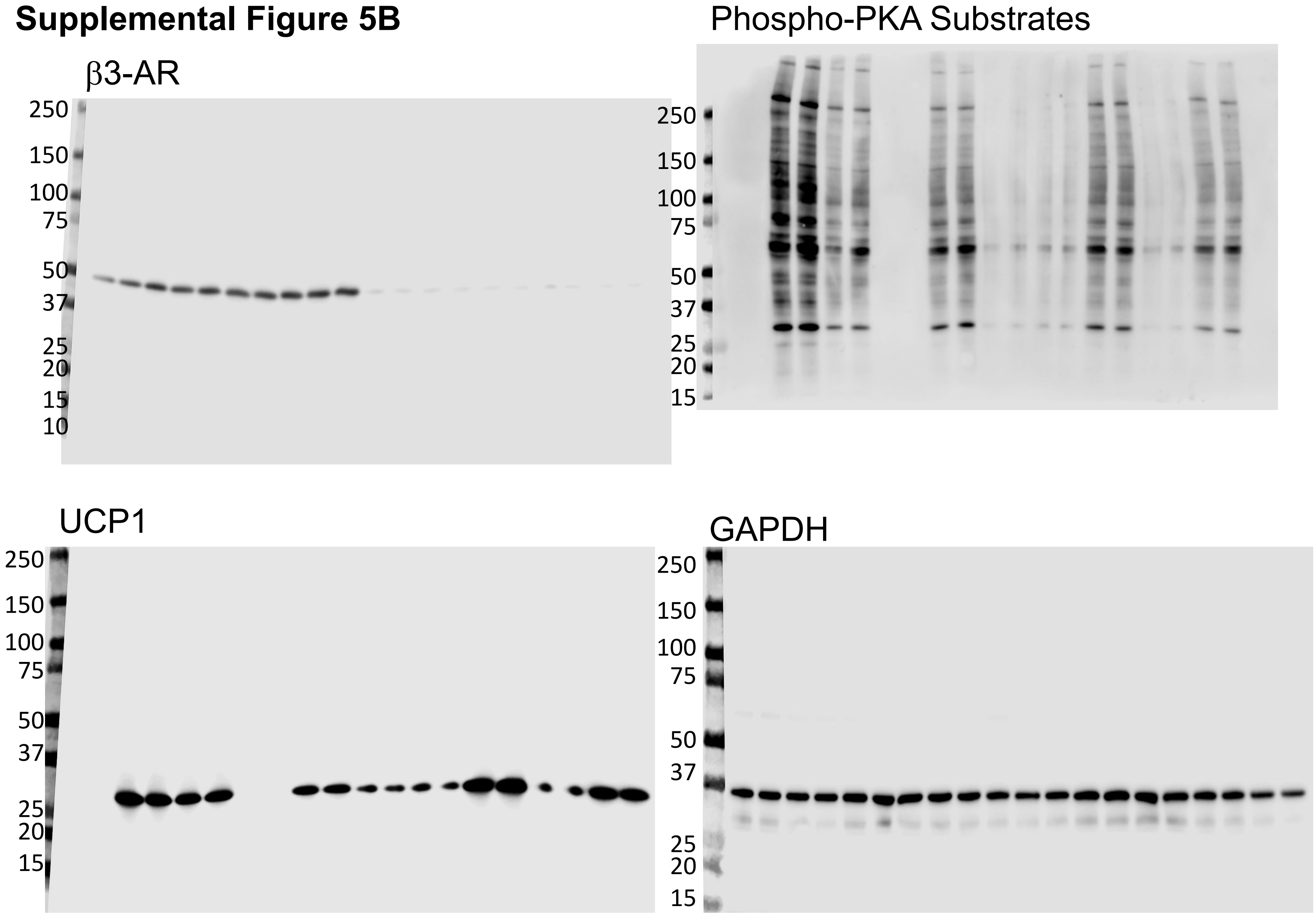

Supplement: Unedited blot and gel images [file jci-134-177726-s136.pdf]
